# The Role of Angiotensin-Converting Enzyme (ACE) Polymorphisms in the Risk of Development and Treatment of Diabetic Nephropathy

**DOI:** 10.3390/jcm13040995

**Published:** 2024-02-08

**Authors:** Magdalena Król-Kulikowska, Nikita Abramenko, Milan Jakubek, Mirosław Banasik, Marta Kepinska

**Affiliations:** 1 Department of Pharmaceutical Biochemistry, Faculty of Pharmacy, Wroclaw Medical University, Borowska 211a, 50-556 Wroclaw, Poland; magdalena.krol-kulikowska@umw.edu.pl; 2 BIOCEV, First Faculty of Medicine, Charles University, 252 50 Vestec, Czech Republic; nikita.abramenko@lf1.cuni.cz (N.A.); milan.jakubek@lf1.cuni.cz (M.J.); 3 Department of Paediatrics and Inherited Metabolic Disorders, First Faculty of Medicine, Charles University and General University Hospital, 120 00 Prague, Czech Republic; 4 Department and Clinic of Nephrology and Transplantation Medicine, Faculty of Medicine, Wroclaw Medical University, Borowska 213, 50-556 Wroclaw, Poland; miroslaw.banasik@umw.edu.pl

**Keywords:** diabetes nephropathy, kidney transplant, single nucleotide polymorphisms, ACE inhibitors, molecular docking

## Abstract

Background: Angiotensin-converting enzyme (ACE) is responsible for the production of angiotensin II, and increased production of angiotensin II is observed in diabetes. What is more, *ACE* polymorphisms may play a role in the development of diabetic nephropathy. The aim of this study was to assess the role of selected *ACE* polymorphisms (rs4343 and rs4646994) in the risk of development of diabetic nephropathy and in the likelihood of renal replacement therapy. Methods: *ACE* polymorphisms were analyzed in a group of 225 patients who were divided into three subgroups. The rs4343 polymorphism was determined using the PCR-RFLP, and the rs4646994 polymorphism was determined using the PCR. Molecular docking between domains of ACE and its ligands was performed by using AutoDock Vina. Results: The G/G genotype of rs4343 polymorphism is associated with increased odds of developing diabetic nephropathy. The G allele is also associated with a higher risk of this disease. Similar results were obtained in patients who had already had a kidney transplant as a result of diabetic nephropathy. Conclusions: The presence of G/G and G/A genotypes, and the G allele increases the likelihood of developing diabetic nephropathy. This may also be a risk factor for renal replacement therapy.

## 1. Introduction

Diabetic nephropathy is one of the most common complications of type 1 and type 2 diabetes, often necessitating kidney transplantation [1,2]. Its pathogenesis is complex, with increasing attention focusing on the role of genetic polymorphisms in its development. In this context, the angiotensin-converting enzyme gene (*ACE*) polymorphisms have garnered particular interest [3,4,5]. ACE is responsible for the production of angiotensin II, a key component of the renin-angiotensin system that plays a crucial role in blood pressure homeostasis by constricting blood vessels [6]. In diabetic individuals, there is a continuous growth in angiotensin II production, leading to elevated oxidative stress, glomerular hyperfiltration, endothelial damage, thrombosis, inflammation, and vascular remodeling [7]. Some of the *ACE* polymorphisms that may be associated with the development of diabetic nephropathy are rs4343 and rs4646994. The rs4343 polymorphism is located in exon 17 of *ACE*. It belongs to single nucleotide polymorphisms (SNPs) and consists of replacing guanine with adenine [8]. However, this does not affect the change in the amino acid sequence. In turn, the rs4646994 polymorphism is located in intron 16 of *ACE*. It is due to the presence of the insertion allele or absence allele of a 287 bp Alu repeat sequence [9]. Ismail et al. [10] observed a more frequent occurrence of the DD genotype within the *ACE* polymorphism (rs4646994) and the D allele within the I/D polymorphism in patients with diabetic nephropathy compared to those with diabetes mellitus but without nephropathy [10]. 

ACE inhibitors (ACEi) are widely used as standard therapy in patients with diabetic nephropathy due to their reported renal protective effects [11]. However, the response to ACEi treatment varies among patients, often being unpredictable, partly due to genetic factors. The contribution of genetics to treatment response differences is primarily associated with the presence of polymorphisms, including single nucleotide polymorphisms (SNPs), insertions/deletions, and variable numbers of tandem repeats (VNTRs) [12,13]. Among the commonly used antihypertensive drugs for diabetic nephropathy treatment are ACEi, such as captopril, lisinopril, or ramipril [14].

The primary mechanism of ACEi action is to inhibit the conversion of angiotensin I to angiotensin II. ACE contains two homologous catalytic domains, the N and C domains, which are capable of cleaving angiotensin I and bradykinin [15,16]. The C domain of ACE is more effective in cleaving angiotensin I to vasoactive angiotensin II [17]. The rs4646994 polymorphism in *ACE* causes premature codon termination, resulting in the enzyme having only one active site in the N domain, thereby limiting drug binding to a single site. In silico analysis is used to visualize and analyze the binding of individual ACEi drugs to these domains [12]. In patients with COVID-19, proteolytic enzymes may selectively affect ACE domains, leading to variable enzyme activity based on the rs4646994 genotype [13]. This is because the genotype within this polymorphism determines how many domains an ACE molecule will have.

The aim of this study was to assess the frequency of two selected *ACE* polymorphisms (rs4343 and rs4646994) in patients with diabetic nephropathy, both with and without kidney transplantation. Additionally, the study aimed to investigate the relationship between specific genotypes, ACE activity, and the concentrations of ACE, creatinine, and C-reactive protein (CRP) in blood serum, as well as glucose in blood plasma. Furthermore, the concentrations of zinc and copper in the serum were determined due to potential disturbances in the metabolism of trace elements like zinc or copper that may occur during the development of diabetic complications [18,19]. The study also explored in silico analysis of interactions between the two ACE domains (N-domain and C-domain) and selected ACE inhibitors (lisinopril, ramipril, enalapril, benazepril).

## 2. Materials and Methods

### 2.1. Study Groups

A total of 225 individuals participated in this study, comprising three groups: the diabetic nephropathy group (*N* = 81), the kidney transplant diabetic nephropathy group (*N* = 94) and the control group (*N* = 50). Biological samples were collected from the participants, including blood samples obtained from Łukasiewicz PORT—Polish Center for Technology Development (control group), and blood samples obtained from the Department and Clinic of Nephrology and Transplantation Medicine of the Wroclaw Medical University (diabetic nephropathy group and kidney transplant diabetic nephropathy group). Blood was collected into two tubes: one tube with clotting activators (to obtain serum; cat. No.: BD 368815, Becton Dickinson, Franklin Lakes, NJ, USA) and the other tube with EDTA (to obtain plasma and buffy coat; cat. No.: BD 367864, Becton Dickinson, USA). DNA was isolated from the buffy coat using a ready-made isolation kit (Syngen Blood/Cell DNA Mini Kit, cat. No.: SY221012, Syngen Biotech, Wrocław, Poland).

The control group consisted of individuals with excluded cardiovascular diseases, liver function disorders (measured by GGT activity, ALT, and ASP), atherosclerosis, diabetes (based on insulin and fasting glucose measurements), hypertension (blood pressure measurements), inflammation (C-reactive protein concentration) and tumors. Potential participants using medications or dietary supplements within the last 6 months were excluded from the study.

The selection of patients was made on the basis of medical history, laboratory tests, and imaging tests (e.g., USG) to exclude other causes of kidney damage. The following parameters were measured in patients: creatinine, blood morphology, urine general examination (including the presence of protein), albuminuria, sodium/potassium, glucose, and GFR (calculated according to the abbreviated formula MDRD). Qualification for the study required the presence of diabetes, albuminuria, proteinuria, or increased creatinine levels. Patients with other causes of kidney damage were excluded. In addition, patients completed a questionnaire providing information such as age, gender, anthropometric data (weight, height), other chronic diseases, stimulant usage (smoking, alcohol consumption), or medications (Questionnaire S1, Supplementary Materials). All participants were informed about the research objectives and provided written consent for the collection of biological material. The Bioethics Committee at Wroclaw Medical University approved the use of collected biological material for research purposes (No. KB 835/2021). The sample size was determined by power analysis using preliminary data from previous studies, with assumptions of α = 0.05 and a power of 80%. 

The characteristics of the three studied groups are presented in Table 1. In order to characterize the groups, the following parameters were used: age, sex, BMI values, glucose and creatinine concentrations, GFR values, and CRP concentrations.

### 2.2. Methods

#### 2.2.1. Determination of ACE Activity, and ACE, Glucose, Creatinine, eGFR, and CRP Concentrations

Serum ACE activity was measured using the ACE1 Activity Assay Kit (Colorimetric) (cat. No.: ab273308, Abcam, Cambridge, UK). Serum ACE concentration was measured using the Human ACE (Angiotensin I Converting Enzyme) ELISA Kit (cat. No.: EH0026, Fine Biotech Co., Ltd., Wuhan, China). Glucose, creatinine, and CRP concentrations were measured in the hospital laboratory during routine patient visits. eGFR values were calculated according to the abbreviated MDRD formula.

#### 2.2.2. Determination of Metal Concentrations

Zinc (Zn) and copper (Cu) concentrations in the blood serum were determined using the SOLAAR M6 atomic absorption spectrophotometer (Thermo Elemental Solaar House, Cambridge, UK) at the Laboratory of Atomic Absorption Spectrometry, Department and Clinic of Internal Diseases, Vocational, Hypertension and Clinical Oncology, Wroclaw Medical University. The Flame Atomic Absorption Spectrometry (FAAS) method in an air-acetylene flame was used to measure the concentrations of these metals.

#### 2.2.3. Genotyping Analysis

DNA was isolated from the buffy coat using the Syngen Blood/Cell DNA Mini Kit (cat. No.: SY221012, Syngen Biotech, Wrocław, Poland). The rs4343 polymorphism was determined using the polymerase chain reaction and restriction fragment length polymorphism analysis (PCR-RFLP). In turn, the rs4646994 polymorphism, due to the fact that it is an insertion/deletion polymorphism, was determined using the polymerase chain reaction (PCR). Primers were designed with the Primer-BLAST program based on gene sequences from GenBank (National Center for Biotechnology Information). The sequences of the primers, reaction conditions, and the restriction enzyme used are presented in Table 2.

The digested DNA fragments were visualized using a 2% agarose gel with Green DNA Gel Stain (both from Syngen Biotech, Wrocław, Poland, with cat. no SY 521011 and cat. no SY 521031, respectively). Electropherograms showing restriction digest products are provided in Figures S1 and S2 (Supplementary Materials).

#### 2.2.4. Molecular Docking

For the docking calculations, three-dimensional (3D) crystallographic structures of the N domain and the C domain of the ACE molecule were obtained from the Protein Data Bank (PDB) with PDB entries 5AMB and 6H5W for N-domain and C-domain, respectively [20]. Prior to the docking procedure, the 3D models were manually prepared to ensure accuracy by removing crystallographic waters, ligands, and other unfavorable components. UCSF Chimera software (version 1.15) was utilized for this purpose [21]. Atoms with double conformations were checked and repaired with a self-written script in Python programming language (version Python 3.8). 

Three-dimensional structures of ligands (benazepril, enalapril, lisinopril, and ramipril) were retrieved from the PubChem open chemistry database using UCSF Chimera software for its downloading. Molecular docking calculations between two domains of ACE and its ligands were performed with AutoDock Vina software (version 1.1.2) [22,23]. The calculations were carried out using the parameters recommended in the user manual. AutoDockTools (ADT, version 1.5.7) was employed to find and determine the center and size of the grid box for the docking calculations. 

The predicted binding affinity (kcal/mol) was calculated by Auto-Dock Vina. To visualize molecules and analyze the docking results, three pieces of software were employed. To generate overall views of the docking outcomes, UCSF Chimera was used [21], while BIOVIA Discovery Studio Visualizer (version 21.1.0.20298) was utilized to create two-dimensional diagrams and illustrate the interactions of ligands with amino acids [24]. PyMOL (version 2.5.2) was employed to verify the positioning of the ligand on the receptor surface. The choice of drugs was made based on an interview with patients who were included in this study. These substances appeared in the drugs used by the respondents.

#### 2.2.5. Statistical Analysis

Statistical analyses were performed using the STATISTICA 13.3 package (Statsoft Polska, Sp. z o.o., Kraków, Poland) under the Wroclaw Medical University license. The normality of variable distributions was assessed using the Shapiro–Wilk test and the homogeneity of variance was examined using Levene’s test.

For testing statistically significant differences between the two groups, the parametric Student’s *t*-test was applied to variables with a normal distribution. If the variable did not meet the conditions of a normal distribution, the non-parametric Mann–Whitney U test was used.

To test statistically significant differences among three or more groups, the non-parametric Kruskal–Wallis test was employed in case the variables did not follow a normal distribution.

The frequencies of genotypes were compared using the χ^2^ test and Fisher’s exact test.

Logistic regression analysis was performed to assess the significance of the effect of polymorphism genotypes on the risk of diabetic nephropathy and the likelihood of renal replacement therapy, expressed as odds ratios (OR) with a 95% confidence interval (CI). Statistical significance was considered for *p* < 0.05.

## 3. Results

### 3.1. Concentrations of the Selected Parameters and ACE Activity in the Studied Groups

Higher ACE concentrations were observed in patients with diabetic nephropathy (*p* = 0.012) and in patients with diabetic nephropathy after kidney transplantation (*p* = 0.005) compared to the control group. In turn, in the case of ACE activity, an inverse relationship was observed (*p* < 0.001 and *p* = 0.003, respectively).

For zinc, the group of patients with diabetic nephropathy showed a lower concentration of this element compared to the control group (*p* < 0.001), while the group of patients after kidney transplantation exhibited a higher concentration of zinc compared to the control group (*p* < 0.001). No significant differences in copper concentrations were found when compared to patients with diabetic nephropathy and patients with diabetic nephropathy after kidney transplantation. The results are presented in Table 3.

### 3.2. The Influence of the rs4343 and the rs4646994 Polymorphisms in ACE on the Concentrations of the Selected Parameters and on ACE Activity

Significant differences in genotypic distribution between the study groups were observed for the rs4343 polymorphism (*p* < 0.001). The G/A genotype appeared least frequently in the control group (8.16%), whereas in the other groups, it was the dominant genotype (50.00% and 46.24%, respectively). Although no similar relationship was observed for the rs4646994 polymorphism, the differences in genotypic distribution were on the verge of statistical significance (*p* = 0.056). The results are presented in Table 4.

#### 3.2.1. The Influence of the rs4343 Polymorphism on the Concentrations of the Selected Parameters and on ACE Activity

After subgrouping the population by genotype (rs4343 polymorphism), no differences in ACE concentration were observed (*p* = 0.118). However, statistically lower ACE activity was observed in the group of patients with diabetic nephropathy and the A/A genotype compared to the control group with the same genotype (*p* = 0.004).

No significant differences were observed between creatinine levels (*p* = 1.000), CRP levels (*p* = 1.000), and copper levels (*p* = 0.485), as well as eGFR values (*p* = 1.000) (Table 5). However, statistically significant differences in glucose concentration were noted. Patients with diabetic nephropathy and the A/A genotype had significantly higher glucose concentrations compared to controls with the A/A genotype (*p* < 0.001). In the case of kidney transplant diabetic nephropathy patients, statistically higher glucose concentrations were observed in the following groups: with the G/G genotype compared to controls with G/G (*p* = 0.009).

Moreover, differences in zinc concentrations were also observed. Patients with diabetic nephropathy and the A/A genotype had lower zinc concentrations compared to the control group with the A/A genotype (*p* = 0.021). On the other hand, kidney transplant diabetic nephropathy patients with the A/A genotype had lower zinc concentrations compared to the control group with the A/A genotype (*p* < 0.001). 

The results described above are presented in Table 5.

#### 3.2.2. The Influence of the rs4646994 Polymorphism on the Concentrations of the Selected Parameter and on ACE Activity

After subgrouping the population by genotype of rs4646994 polymorphism, no significant differences were observed between creatinine levels (*p* = 1.000), eGFR values (*p* = 1.000), or copper levels (*p* = 0.645). However, in the case of glucose concentration, the following observations were noted: patients with diabetic nephropathy and patients with the I/D genotype after kidney transplantation had higher glucose concentrations than the control group with the same genotype.

For CRP levels, patients with the I/D genotype (p = 0.003) after kidney transplantation had statistically higher CRP levels compared to the control group with the I/D genotype. 

Regarding zinc concentrations, statistically significant lower concentrations of this element were observed in the following groups: patients with diabetic nephropathy and the I/D compared to the control group with the same genotype (*p* < 0.001), as well as patients after kidney transplantation and the I/D genotype compared to the control group with the same genotype (*p* < 0.001).

The results described above are presented in Table 6.

#### 3.2.3. The Influence of the rs4646994 Polymorphism on the Concentration of Selected Parameters and ACE Activity in a Group of Patients Using Ramipril

Some of the patients were treated with ACEi. They most often took ramipril (31 patients); they also used perindopril (9 patients), lisinopril (3 patients), and quinapril (1 patient). Due to the small groups, a full statistical analysis of the results was not performed. However, Table 7 shows the relationship between the rs4646994 polymorphism and the values of selected parameters in the group of patients using ramipril. Statistically higher glucose levels were observed in patients with the I/D genotype compared to patients with the I/I genotype (*p* = 0.027).

### 3.3. The Influence of ACE Polymorphisms on the Risk of Occurrence of Diabetic Nephropathy or the Likelihood of Renal Replacement Therapy

In this study, logistic regression was used to assess the risk of developing diabetic nephropathy or the likelihood of renal replacement therapy based on *ACE* polymorphisms. The results indicate that the G/G genotype (rs4343 polymorphism) is associated with an over 2.68-fold increased odds of developing diabetic nephropathy (*p* = 0.014). Another genotype within this polymorphism, G/A, also seems to be associated with a significantly increased risk of developing this complication of diabetes. However, the wide confidence interval suggests low accuracy in estimating this parameter. Nevertheless, the occurrence of the G allele is associated with a 2.53-fold higher risk of developing nephropathy (*p* < 0.001). Additionally, each subsequent year increases the risk of developing diabetic nephropathy by 17.30% (*p* < 0.001), while an increase in BMI by one unit increases this risk by 26.60% (*p* < 0.001).

Similar results were obtained in the kidney transplant diabetic nephropathy group. The G/G and G/A genotypes within the rs4343 polymorphism were associated with an increased likelihood of renal replacement therapy (approximately 3.35-fold and 15.23-fold, respectively). Likewise, the G allele was associated with a 2.89-fold increased likelihood of renal replacement therapy. Additionally, each subsequent year was associated with a 1.18-fold decreased likelihood of renal replacement therapy (*p* < 0.001), while an increase in BMI by one unit was associated with a 1.21-fold decreased likelihood (*p* < 0.001). 

The results described above are presented in Table 8 and Table 9.

### 3.4. Interaction of ACE with Selected Drugs (Benazepril, Enalapril, Lisinopril and Ramipril)

**Figure 1:**
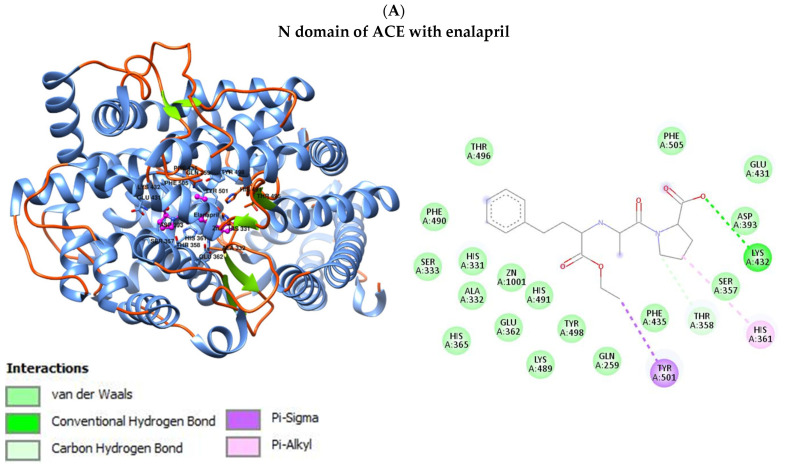

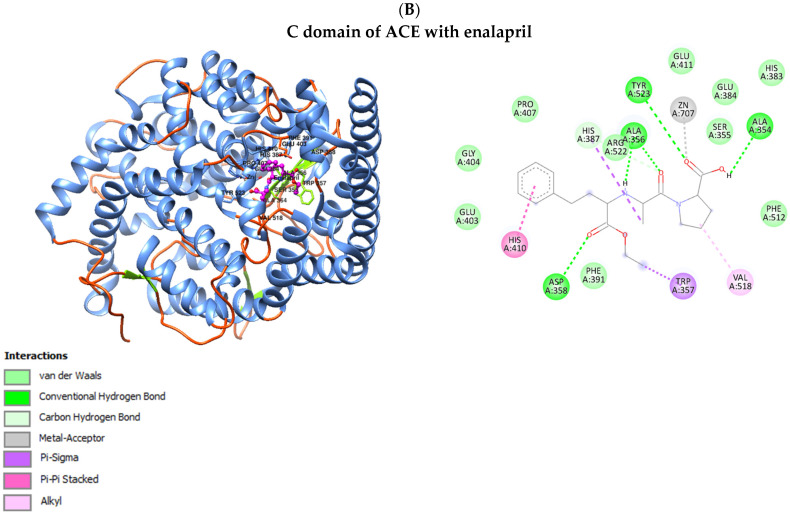
The interaction between N and C domains of ACE and enalapril.

The molecular docking analysis was performed to calculate the binding affinity between the N and C domains of ACE and their ligands—drugs from the ACEi group, which are used in the treatment of diabetic nephropathy. The obtained results indicate that enalapril and ramipril have similar binding affinities for both domains. This means that the two drugs effectively bind to both the N and the C domains. In turn, benazepril was noted to have a lower binding affinity to the C domain compared to the N domain. This indicates that benazepril binds more efficiently with the C domain. A similar relationship was observed with lisinopril, which also showed a lower binding affinity to the C domain compared to the second domain. The discussed results are shown in Table 10. The interaction of enalapril between the N and C domains of ACE is shown in Figure 1. The visualization of interactions with ramipril, benazepril and lisinopril are presented in the Supplementary Materials (Figures S3–S5).

## 4. Discussion

Diabetic nephropathy is the leading cause of mortality in diabetic patients [25]. Emerging evidence points to the importance of the role played by the ACE molecule in the pathogenesis of diabetic nephropathy [26,27]. Although there are indications that identify *ACE* polymorphisms as one of the risk factors in kidney transplant rejection [28,29], there are no studies that would confirm their role in the development of diabetic nephropathy that leads to transplantation. The current study was undertaken to investigate polymorphisms in the *ACE* gene (rs4343 and rs4646994) in patients with diabetic nephropathy (without and after transplantation), as well as to compare the binding of ACEi drugs used in nephrology with two ACE domains.

The studied polymorphisms (rs4343 and rs4646994) had no effect on ACE concentrations. However, it was noticed that ACE activity in the group of patients with diabetic nephropathy and the A/A genotype was significantly lower compared to the control group. This is interesting because the situation was different in the case of the concentrations of this enzyme—patients from the control group had higher concentrations of this parameter. Low ACE activity in patients with diabetic nephropathy may result from compensatory adaptation aimed at reducing the production of additional angiotensin II (Ang II) [30,31]. 

This study also demonstrated a relationship between zinc concentrations and the occurrence of diabetic nephropathy. Lower concentrations of this element were observed in patients with diabetic nephropathy compared to the control group, which corresponds to the previously available data [32,33]. A low supply of zinc in the diet and its low concentrations in blood serum are associated with an increased incidence of diabetes and cardiovascular diseases. Unfortunately, it is not always known whether the disease affects zinc metabolism or whether its low concentrations result in carbohydrate metabolism disorders. It is highly probable that these two phenomena coexist [32]. What is interesting is that patients after kidney transplantation had higher zinc concentrations compared to the control group, which would be contrary to other studies [34,35]. However, after a more detailed analysis of the groups, it turned out that these groups of patients with the A/A (rs4343) and D/D (rs4646994) genotypes had lower zinc concentrations compared to the corresponding control groups. Perhaps this discrepancy is caused by the fact that the results of the statistical test comparing the three groups (the control group, the diabetic nephropathy group, and the kidney transplant diabetic nephropathy group) were influenced by both very low and very high zinc concentrations in the kidney transplant diabetic nephropathy group. In turn, after additional division of this group due to different genotypes, the concentrations of the tested element were distributed slightly differently between the individual groups. However, in the case of copper, no statistical significance was observed. 

The influence of genetic factors, including two *ACE* polymorphisms, on an increased risk of developing diabetic nephropathy or an increased likelihood of renal replacement therapy due to ongoing diabetic nephropathy has been investigated. Based on the logistic regression results, it can be seen that the rs4343 polymorphism could be a useful tool in predicting this risk in both cases. Both genotypes containing the G allele (G/G and G/A) and the G allele alone were associated with an increased risk of developing diabetic nephropathy or an increased likelihood of renal replacement therapy. These results are confirmed by other studies, which also indicate the importance of the G allele (rs4343 polymorphism) in the pathogenesis of diabetic complications [36,37]. In order to use the obtained results in clinical terms, cohort studies should be conducted, taking into account the interaction of the studied polymorphism with other gene variants. Similar dependencies were not obtained in the case of the rs4646994 polymorphism, the influence of which was confirmed in many research reports [38,39,40]. This may be due to the fact that studies were carried out on groups not exceeding 100 patients, so in order to confirm these results, a much larger number of patients with diabetic nephropathy should be examined. Sex also does not seem to be a significant risk factor in the development of the studied disease, which would be consistent with the results obtained by other researchers [41]. However, older age and higher BMI values have been found to increase the risk of developing diabetic nephropathy. This corresponds to the current knowledge, according to which obesity is one of the factors of diabetes development and contributes to its complications [42,43].

Although in silico studies in the context of the impact of the rs4646994 polymorphism on treatment with ACEi drugs have already been conducted, their main target was usually captopril or lisinopril [44,45]. In this study, the interactions between two ACE domains and four drugs that are registered in Poland as those that can be used in the treatment of diabetic nephropathy [46] were taken into account. It should be remembered here that the I allele of the rs4646994 polymorphism causes premature codon termination, resulting in the enzyme having only one active site in the N domain [12]. This means that potential drugs are able to attach to only one active site, which may affect their effectiveness. In addition, it has been proven that different drugs bind to particular domains, showing different binding affinities [12,15,16,17]. In the present study, it was observed that benazepril and lisinopril display a significant preference for the C domain of the ACE, although the calculated value for the N domain was still relevant. Nevertheless, enalapril and ramipril can bind with very similar efficiency to both domains; therefore, the rs4646994 polymorphism should not have a significant impact on their action in the treatment of diabetic nephropathy. Moreover, after comparing the concentrations of selected parameters in groups based on ramipril use and genotypes, no significant differences were observed. The exception was the glucose concentration, which was higher in the group of patients with the I/D genotype compared to patients with the I/I genotype. However, it should be taken into account that a relatively small group of patients took ramipril; therefore, a larger study group should be used to examine the relationship between the drugs used and the selected genotypes. Therefore, in the case of the other two drugs—benazepril and lisinopril—the genotype within the rs4646994 polymorphism may translate into the effectiveness of these agents. Both drugs bind more effectively to the C domain, so in patients with the I/D and I/I genotypes, the effectiveness of benazepril and lisinopril may be lower compared to patients with the D/D genotype. This could translate into the clinical effectiveness of these drugs, but in order to confirm the obtained results, direct tests of the effectiveness of individual ACEi should be carried out in groups of patients divided according to the genotype within the rs4646994 polymorphism.

## 5. Conclusions

In conclusion, the study reveals significant associations between one of the *ACE* polymorphisms (rs4343) and the risk of diabetic nephropathy or the likelihood of renal replacement therapy. The presence of specific genotypes (G/G and G/A) and G alleles increases the likelihood of developing these complications. Additionally, age and BMI were identified as factors influencing the risk of diabetic nephropathy, while sex did not show a significant association. Similar results were obtained to investigate the likelihood of renal replacement therapy.

However, it is important to remember that not only the above-mentioned factors will contribute to the increased risk of renal replacement therapy. Patients who develop diabetic nephropathy and have it for a long time will most likely require this type of therapy in the future. Therefore, it would be appropriate to investigate the impact of *ACE* polymorphisms on the risk of kidney transplant rejection. In addition, extended studies could take into account the impact of selected *ACE* polymorphisms on the development of diabetic nephropathy by assessing the progression of renal failure using the speed of the decline of eGFR. Additional tests should also be performed, such as the assessment of proteinuria as an important factor in the progression of renal failure.

Moreover, the results of in silico analysis seem to be interesting. They indicate the dependence of the effectiveness of treatment with ACEi on the rs4646994 polymorphism. However, it should be noted that these are only preliminary studies. To confirm these results, clinical trials should be conducted on a larger group of patients. Obtaining similar results could contribute to the individualization of therapy and, thus, more effective treatment.

## Figures and Tables

**Table 1:** Values and concentrations of selected parameters characterizing the studied groups.

Parameter	Control Group (*N* = 50)	Diabetic Nephropathy Group (*N* = 81)	Kidney Transplant Diabetic Nephropathy Group (*N* = 94)	*p*
Age (years)	{25; 34; 47}	{65; 71; 78} *	{55; 62; 69} *^,^**	<0.001
Sex	Men: 21 Women: 29	Men: 42 Women: 39	Men: 47 Women: 47	0.540
BMI (kg/m^2^)	23.83 ± 3.37	30.02 ± 5.33 *	27.05 ± 4.76 *^,^**	<0.001
Glucose (mg/dL)	{81.00; 85.50; 88.92}	{106.00; 139.50; 178.00} *	{113.00; 139.50; 173.00} *	<0.001
Creatinine (mg/dL)	-	{1.14; 1.36; 1.72}	{1.14; 1.30; 1.70}	0.405
eGFR (mL/min/1.73 m^2^)	-	{35.00; 48.00; 58.00}	{42.00; 53.50; 63.00}	0.086
CRP (mg/L)	{0.33; 0.65; 1.10}	{0.72; 1.91; 4.88} *	{0.97; 2.66; 4.11} *	<0.001

Values are shown as mean value ± standard deviation or {1st quartile; median; 3rd quartile}. * *p* < 0.05—compared to control group; ** *p* < 0.05—compared to diabetic nephropathy group.

**Table 2:** The conditions for PCR and restriction enzyme digestion.

SNP	Primers	PCR-RFLP Conditions
rs4343	Forward primer—5′ CTG ACG AAT GTG ATG GCC GC 3′ Reverse primer—5′ TGA TGA GTT CCA CGT ATT TCG 3′	the initial denaturation—95 °C for 5 min denaturation—95 °C for 40 s annealing—58.4 °C for 35 s elongation—72 °C for 40 s the final elongation—72 °C for 10 min
	**Restriction enzyme**	**Restriction enzyme digestion conditions**
	BstUI	37 °C for 16 h
rs4646994	Forward primer—5′ CTG GAG ACC ACT CCC ATC CTT TCT 3′ Reverse primer—5′ GAT GTG GCC ATC ACA TTC GTC AGA T 3′	the initial denaturation—95 °C for 5 min denaturation—95 °C for 40 s annealing—60 °C for 35 s elongation—72 °C for 40 s the final elongation—72 °C for 10 min

**Table 3:** Concentration of ACE, activity of ACE, and concentrations of zinc and copper in the studied groups.

Parameter	Control Group (*N* = 50)	Diabetic Nephropathy Group (*N* = 81)	Kidney Transplant Diabetic Nephropathy Group (*N* = 94)	*p*
ACE (ng/mL)	{42.64; 71.97; 99.30}	{79.06; 89.52; 101.72} *	{81.25; 89.64; 100.38} *	0.005
ACE (mU/mL)	{0.066; 0.079; 0.092}	{0.026; 0.052; 0.071} *	{0.045; 0.063; 0.076} *	<0.001
Zn (µg/L)	{755.00; 830.00; 913.50}	{720.00; 804.50; 880.00} *	{854.21; 946.55; 1031.87} *	<0.001
Cu (µg/L)	{880.00; 1019.00; 1151.50}	{909.00; 1084.00; 1200.00}	{920.91; 1012.89; 1181.65}	0.293

Values are shown as {1st quartile; median; 3rd quartile}. * *p* < 0.05—compared to the control group.

**Table 4:** The genotypic distribution of the rs4343 and the rs4646994 polymorphisms of *ACE* in the studied groups.

SNP	Groups (N)	Genotype Frequencies (%)		
		G/G	G/A	A/A
rs4343	Control (*N* = 49)	*N* = 11 (22.45%)	*N* = 4 (8.16%)	*N* = 34 (69.39%)
	Diabetic Nephropathy (*N* = 84)	*N* = 16 (19.05%)	*N* = 42 (50.00%)	*N* = 26 (30.95%)
	Kidney Transplant Diabetic Nephropathy (*N* = 93)	*N* = 26 (27.96%)	*N* = 43 (46.24%)	*N* = 24 (25.80%)
**SNP**	**Groups (*N*)**	**Genotype Frequencies (%)**		
		**I/I**	**I/D**	**D/D**
rs4646994	Control (*N* = 49)	*N* = 0 (0.00%)	*N* = 42 (84.00%)	*N* = 8 (16.00%)
	Diabetic Nephropathy (*N* = 81)	*N* = 11 (13.58%)	*N* = 51 (62.96%)	*N* = 19 (23.46%)
	Kidney Transplant Diabetic Nephropathy (*N* = 93)	*N* = 13 (13.98%)	*N* = 59 (63.44%)	*N* = 21 (22.58)

**Table 5:** Concentrations and activity of the selected parameters in the studied groups in terms of the rs4343 polymorphism in *ACE*.

Parameter	Control Group (*N* = 49)			Diabetic Nephropathy Group (*N* = 84)			Kidney Transplant Diabetic Nephropathy Group (*N* = 93)		
	G/G (*N* = 11)	G/A (*N* = 4)	A/A (*N* = 34)	G/G (*N* = 16)	G/A (*N* = 42)	A/A (*N* = 26)	G/G (*N* = 26)	G/A (*N* = 43)	A/A (*N* = 24)
ACE (ng/mL)	{67.86; 83.77; 88.92}	{51.04; 60.85; 90.94}	{41.62; 78.13; 101.64}	{81.66; 88.36; 104.25}	{79.39; 90.71; 100.17}	{77.10; 86.63; 101.97}	{79.55; 84.74; 97.05}	{81.02; 90.20; 101.21}	{85.90; 94.63; 100.64}
ACE (mU/mL)	{0.073; 0.078; 0.106}	{0.079; 0.085; 0.088}	{0.063; 0.079; 0.093}	{0.050; 0.063; 0.077}	{0.022; 0.049; 0.074}	{0.025; 0.051; 0.066} **	{0.054; 0.063; 0.073]	{0.040; 0.063; 0.076}	{0.037; 0.064; 0.078}
Glucose (mg/dL)	{81.00; 82.98; 88.92}	{85.50; 90.00; 94.50}	{79.92; 84.47; 88.92}	{102.00; 124.50; 154.00}	{106.00; 139.00; 183.00}	{105.00; 141.00; 163.00} **	{114.00; 126.00; 143.00} *	{111.00; 149.00; 184.00}	{113.00; 155.00; 183.00}
Creatinine (mg/dL)	-	-	-	{1.06; 1.35; 1.46}	{1.21; 1.42; 2.00}	{1.05; 1.24; 1.60}	{1.08; 1.26; 1.41}	{1.14; 1.31; 1.80}	{1.17; 1.30; 1.70}
eGFR (mL/min/1.73 m^2^)	-	-	-	{48.50; 52.00; 59.50}	{29.00; 44.00; 54.00}	{44.00; 56.00; 64.00}	{43.00; 56.00; 61.00}	{36.00; 53.00; 63.00}	{45.00; 54.00; 66.00}
CRP (mg/L)	{0.27; 0.64; 1.01}	{0.37; 0.99; 3.12}	{0.33; 0.65; 1.10}	{0.60; 2.55; 4.49}	{1.29; 2.38; 5.35}	{0.23; 0.72; 1.24}	{1.21; 2.79; 4.07}	{1.49; 2.26; 4.33}	{0.70; 0.96; 4.10}
Zn (µg/L)	{845.49; 895.37; 1019.99}	{823.33; 933.47; 1075.62}	{901.82; 972.73; 1043.34}	{817.00; 855.00; 922.00}	{752.00; 800.50; 884.00}	{729.00; 855.50; 937.00} **	{749.00; 825.00; 940.00}	{721.00; 807.00; 887.00}	{672.00; 753.50; 833.00} **
Cu (µg/L)	{986.90; 1046.20; 1184.39}	{1006.96; 1251.14; 2039.81}	{868.53; 998.18; 1155.12}	{896.00; 1009.00; 1205.00}	{884.00; 1002.00; 1168.00}	{859.00; 1058.00; 1129.00}	{904.00; 1047.00; 1158.00}	{911.00; 1092.00; 1254.00}	{900.00; 1074.00; 1225.00}

Values are shown as {1st quartile; median; 3rd quartile}. * *p* < 0.05—compared to the control group with the G/G genotype; ** *p* < 0.05—compared to the control group with the A/A genotype.

**Table 6:** Concentrations of the selected parameters in the studied groups in terms of the rs46464994 polymorphism in *ACE*.

Parameter	Control Group (*N* = 50)			Diabetic Nephropathy Group (*N* = 81)			Kidney Transplant Diabetic Nephropathy Group (*N* = 93)		
	I/I (*N* = 0)	I/D (*N* = 42)	D/D (*N* = 8)	I/I (*N* = 11)	I/D (*N* = 51)	D/D (*N* = 19)	I/I (*N* = 13)	I/D (*N* = 59)	D/D (*N* = 21)
ACE (ng/mL)	-	{42.64; 71.51; 89.32}	{60.50; 88.52; 99.30}	{80.05; 96.22; 101.98}	{76.48; 87.19; 100.17}	{83.22; 88.32; 101.72}	{86.33; 93.61; 96.98}	{80.05; 89.64; 101.02}	{76.15; 86.66; 100.49}
ACE (mU/mL)	-	{0.063; 0.079; 0.092}	{0.074; 0.077; 0.086}	{0.016; 0.036; 0.070}	{0.022; 0.051; 0.074} *	{0.045; 0.057; 0.073}	{0.027; 0.064; 0.069}	{0.040; 0.062; 0.076}	{0.054; 0.063; 0.073}
Glucose (mg/dL)	-	{81.00; 85.50; 88.92}	{81.99; 85.95; 92.52}	{103.00; 110.00; 146.00}	{107.00; 141.00; 183.00} *	{94.50; 152.00; 202.50}	{130.50; 161.00; 178.00}	{113.00; 139.00; 170.00} *	{112.00; 122.00; 146.00}
Creatinine (mg/dL)	-	-	-	{1.12; 1.24; 1.34}	{1.18; 1.39; 1.80}	{1.14; 1.41; 1.48}	{1.12; 1.27; 1.54}	{1.14; 1.41; 1.80}	{1.06; 1.22; 1.31}
eGFR (mL/min/1.73 m^2^)	-	-	-	{54.00; 56.00; 61.00}	{34.00; 45.00; 55.50}	{36.00; 50.00; 61.00}	{45.50; 54.00; 67.00}	{36.00; 51.00; 59.00}	{52.00; 59.00; 64.00}
CRP (mg/L)	-	{0.37; 0.72; 1.15}	{0.15; 0.36; 1.03}	{0.71; 0.98; 1.24}	{0.72; 2.13; 5.42}	{0.60; 1.94; 4.49}	{0.77; 0.93; 4.11}	{0.99; 2.86; 4.33} *	{1.21; 2.43; 3.37}
Zn (µg/L)	-	{890.78; 972.73; 1043.34}	{824.51; 877.31; 995.86}	{762.00; 879.00; 945.00}	{741.00; 807.00; 898.00} *	{759.00; 855.00; 922.00}	{704.00; 744.00; 798.00}	{718.00; 805.00; 887.00} *	{780.00; 825.00; 880.00}
Cu (µg/L)	-	{920.91; 1002.02; 1181.65}	{930.06; 1032.31; 1233.67}	{835.00; 941.00; 1144.00}	{896.00; 1045.00; 1150.00}	{822.00; 962.00; 1205.00}	{820.00; 1097.00; 1225.00}	{911.00; 1087.00; 1248.00}	{934.00; 1088.00; 1139.00}

Values are shown as {1st quartile; median; 3rd quartile}. * *p* < 0.05—compared to the control group with the I/D genotype.

**Table 7:** Concentrations of the selected parameters in the groups of patients using ramipril in terms of the rs46464994 polymorphism in *ACE*.

Parameter	I/I (*N* = 6)	I/D (*N* = 17)	D/D (*N* = 8)	*p*
BMI (kg/m^2^)	{22.54; 32.57; 38.81}	{26.29; 27.92; 33.27}	{29.28; 31.99; 40.39}	0.186
ACE (ng/mL)	{86.48; 92.68; 97.97}	{83.10; 89.61; 104.70}	{81.20; 92.97; 97.35}	0.983
ACE (mU/mL)	{0.027; 0.051; 0.066}	{0.018; 0.062; 0.077}	{0.035; 0.069; 0.073}	0.886
Glucose (mg/dL)	{103.00; 106.00; 110.00}	{133.00; 157.00; 216.00} *	{128.00; 146.50; 224.00}	0.027
Creatinine (mg/dL)	1.23 ± 0.21	1.26 ± 0.34	1.30 ± 0.29	0.919
eGFR (mL/min/1.73 m^2^)	53.67 ± 9.33	61.13 ± 21.36	54.88 ± 17.72	0.620
CRP (mg/L)	{1.31; 2.72; 5.02}	{0.45; 0.72; 1.54}	-	0.395
Zn (µg/L)	778.67 ± 105.56	810.47 ± 150.56	797.50 ± 60.63	0.947
Cu (µg/L)	1007.83 ± 225.69	1067.88 ± 216.30	940.25 ± 188.94	0.386

Values are shown as mean value ± standard deviation or {1st quartile; median; 3rd quartile}. * *p* < 0.05—compared to the I/I group.

**Table 8:** The relationship between the selected parameters and the risk of developing diabetic nephropathy.

SNP (*Gene*)	Genotype	Diabetic Nephropathy Group	Control Group	*p*	OR	95% CI OR
rs4343 (*ACE*)	G/G	42	11	0.014	2.675	1.216–5.884
	G/A	85	4	<0.001	13.894	4.662–41.408
	A/A	50	34	-	1.000	-
	G allele	169	26	<0.001	2.530	1.543–4.148
	A allele	185	72	-	1.000	-
rs4646994 (*ACE*)	I/I	24	0	-	-	-
	I/D	110	42	0.131	0.524	0.227–1.211
	D/D	40	8	-	1.000	-
	I allele	168	42	0.382	1.221	0.780–1.911
	D allele	190	58	-	1.000	-
**Other** **Variables**	**Category**	**Diabetic** **Nephropathy Group**	**Control Group**	***p***	**OR**	**95% CI OR**
Age	-	-	-	<0.001	1.173	1.123–1.226
BMI	-	-	-	<0.001	1.266	1.157–1.386
Sex	Men	89	21	-	1.000	-
	Women	85	29	0.270	0.701	0.372–1.318

**Table 9:** The relationship between the selected parameters and the likelihood of renal replacement therapy.

SNP (*Gene*)	Genotype	Kidney Transplant Diabetic Nephropathy Group	Control Group	*p*	OR	95% CI OR
rs4343 (*ACE*)	G/G	26	11	0.007	3.348	1.392–8.053
	G/A	43	4	<0.001	15.229	4.821–48.103
	A/A	24	34	-	1.000	-
	G allele	95	26	<0.001	2.891	1.697–4.925
	A allele	91	72	-	1.000	-
rs4646994 (*ACE*)	I/I	13	0	-	-	-
	I/D	59	42	0.176	0.535	0.216–1.323
	D/D	21	8	-	1.000	-
	I allele	85	42	0.548	1.162	0.711–1.899
	D allele	101	58	-	1.000	-
**Other** **Variables**	**Category**	**Kidney** **Transplant** **Diabetic** **Nephropathy Group**	**Control Group**	***p***	**OR**	**95% CI OR**
Age	-	-	-	<0.001	0.848	0.805–0.894
BMI	-	-	-	<0.001	0.826	0.748–0.911
Sex	Men	47	21	-	1.000	-
	Women	46	29	0.327	1.409	0.710–2.798

**Table 10:** The binding affinity between two domains of ACE and enalapril, ramipril, benazepril, and lisinopril.

Ligands	The Binding Affinity (kcal/mol)	
	N Domain	C Domain
Enalapril	−7.4	−7.9
Ramipril	−8.0	−8.2
Benazepril	−6.6	−8.3
Lisinopril	−5.8	−6.8

## Data Availability

The data presented in this study are available on request from the corresponding author. The data are not publicly available due to a lack of patients’ consent to making their data public.

## Supplementary Materials

The following supporting information can be downloaded at https://www.mdpi.com/article/10.3390/jcm13040995/s1. Questionnaire S1: Sample of a questionnaire conducted among people suffering from diabetic nephropathy; Figure S1: Example of electropherogram for rs4343 (*ACE*); Figure S2: Example of electropherogram for rs4646994 (*ACE*); Figure S3: The interaction between N and C domains of ACE and ramipril; Figure S4: The interaction between N and C domains of ACE and benazepril; Figure S5: The interaction between N and C domains of ACE and lisinopril.

## References

[1] Madziarska K., Banasik M. (2006). Chorzy na cukrzycę w programach hemodializy i dializy otrzewnowej—Zagrożenia, których można uniknąć [Diabetes patients in hemodialysis and peritoneal dialysis programs—risks that can be avoided]. Probl. Lek..

[2] Sulaiman M.K. (2019). Diabetic nephropathy: Recent advances in pathophysiology and challenges in dietary management. Diabetol. Metab. Syndr..

[3] Rahimi Z. (2012). ACE insertion/deletion (I/D) polymorphism and diabetic nephropathy. J. Nephropathol..

[4] Yu Z.Y., Chen L.S., Zhang L.C., Zhou T.B. (2012). Meta-analysis of the relationship between ACE I/D gene polymorphism and end-stage renal disease in patients with diabetic nephropathy. Nephrology.

[5] Huang Z., Wu B., Tao J., Han Z., Yang X., Zhang L., Liu X., Wang Z., Tan R., Gu M. (2015). Association between Angiotensin I-Converting Enzyme Insertion/Deletion Polymorphism and Prognosis of Kidney Transplantation: A Meta-Analysis. PLoS ONE.

[6] Rahimi Z., Hasanvand A., Felehgari V. (2012). Interaction of MTHFR 1298C with ACE D allele augments the risk of diabetic nephropathy in Western Iran. DNA Cell Biol..

[7] Ruggenenti P., Bettinaglio P., Pinares F., Remuzzi G. (2008). Angiotensin converting enzyme insertion/deletion polymorphism and renoprotection in diabetic and nondiabetic nephropathies. Clin. J. Am. Soc. Nephrol..

[8] Alimoradi N., Sharqi M., Firouzabadi D., Sadeghi M.M., Moezzi M.I., Firouzabadi N. (2022). SNPs of ACE1 (rs4343) and ACE2 (rs2285666) genes are linked to SARS-CoV-2 infection but not with the severity of disease. Virol. J..

[9] Moradzadegan A., Vaisi-Raygani A., Nikzamir A., Rahimi Z. (2015). Angiotensin converting enzyme insertion/deletion (I/D) (rs4646994) and Vegf polymorphism (+405G/C; rs2010963) in type II diabetic patients: Association with the risk of coronary artery disease. J. Renin-Angiotensin-Aldosterone Syst..

[10] Ismail M.F., Shaker O.G., Ashour E., Yousif H.M., Afify M., Gouda W. (2017). The combined effect of ACE, TCF7L2, and PPARGC1A gene polymorphisms in diabetic nephropathy. Egypt. J. Chem..

[11] Aggarwal N., Kare P.K., Varshney P., Kalra O.P., Madhu S.V., Banerjee B.D., Yadav A., Raizada A., Tripathi A.K. (2017). Role of angiotensin converting enzyme and angiotensinogen gene polymorphisms in angiotensin converting enzyme inhibitor-mediated antiproteinuric action in type 2 diabetic nephropathy patients. World J. Diabetes.

[12] Widodo W., Wisnasari S., Saifur Rohman M., Yunita L., Lukitasari M., Nuril M., Holil K., Laila Purwaningroom D. (2017). Alu insertion/deletion of ACE gene polymorphism might not affect significantly the serum bradykinin level in hypertensive patients taking ACE inhibitors. Egypt. J. Med. Hum. Genet..

[13] Papadopoulou A., Fragkou P.C., Maratou E., Dimopoulou D., Kominakis A., Kokkinopoulou I., Kroupis C., Nikolaidou A., Antonakos G., Papaevangelou V. (2022). Angiotensin-converting-enzyme insertion/deletion polymorphism, ACE activity, and COVID19: A rather controversial hypothesis. A case-control study. J. Med. Virol..

[14] Hye Khan M.A., Imig J.D. (2018). Antihypertensive Drugs. Reference Module in Biomedical Sciences.

[15] Ceconi C., Francolini G., Olivares A., Comini L., Bachetti T., Ferrari R. (2007). Angiotensin converting enzyme (ACE) inhibitors have different selectivity for bradykinin binding sites of human somatic ACE. Eur. J. Pharmacol..

[16] Fernandez J.H., Hayashi M.A., Camargo A.C., Neshich G. (2003). Structural basis of the lisinopril-binding specificity in N- and C-domains of human somatic ACE. Biochem. Biophys. Res. Commun..

[17] Denti P., Sharp S.K., Kröger W.L., Schwager S.L., Mahajan A., Njoroge M., Gibhard L., Smit I., Chibale K., Wiesner L. (2014). Pharmacokinetic evaluation of lisinopril-tryptophan, a novel C-domain ACE inhibitor. Eur. J. Pharm. Sci..

[18] Feng J., Wang H., Jing Z., Wang Y., Wang W., Jiang Y., Sun W. (2021). Relationships of the Trace Elements Zinc and Magnesium with Diabetic Nephropathy-Associated Renal Functional Damage in Patients with Type 2 Diabetes Mellitus. Front. Med..

[19] Ming J., Sana S.R.G.L., Deng X. (2022). Identification of copper-related biomarkers and potential molecule mechanism in diabetic nephropathy. Front. Endocrinol..

[20] Berman H.M., Westbrook J., Feng Z., Gilliland G., Bhat T.N., Weissig H., Shindyalov I.N., Bourne P.E. (2000). The Protein Data Bank. Nucleic Acids Res..

[21] Pettersen E.F., Goddard T.D., Huang C.C., Couch G.S., Greenblatt D.M., Meng E.C., Ferrin T.E. (2004). UCSF Chimera—A visualization system for exploratory research and analysis. J. Comput. Chem..

[22] Eberhardt J., Santos-Martins D., Tillack A.F., Forli S. (2021). AutoDock Vina 1.2.0: New Docking Methods, Expanded Force Field, and Python Bindings. J. Chem. Inf. Model..

[23] Trott O., Olson A.J. (2010). AutoDock Vina: Improving the speed and accuracy of docking with a new scoring function, efficient optimization, and multithreading. J. Comput. Chem..

[24] Biovia D.S. (2020). BIOVIA Discovery Studio Visualizer (2D Diagram and Scheme of the Interactions with Amino Acids).

[25] Sagoo M.K., Gnudi L. (2020). Diabetic Nephropathy: An Overview. Methods Mol. Biol..

[26] Kobori H., Kamiyama M., Harrison-Bernard L.M., Navar L.G. (2013). Cardinal role of the intrarenal renin-angiotensin system in the pathogenesis of diabetic nephropathy. J. Investig. Med..

[27] Kim S.S., Kim J.H., Kim I.J. (2016). Current Challenges in Diabetic Nephropathy: Early Diagnosis and Ways to Improve Outcomes. Endocrinol. Metab..

[28] ra Lee S., Moon J.Y., Lee S.H., Ihm C.G., Lee T.W., Kim S.K., Chung J.H., Kang S.W., Kim T.H., Park S.J. (2013). Angiotensinogen polymorphisms and post-transplantation diabetes mellitus in Korean renal transplant subjects. Kidney Blood Press. Res..

[29] Azmandian J., Mohamadifar M., Rahmanian-Koshkaki S., Mehdipoor M., Nematollahi M.H., Saburi A., Mandegary A. (2015). Study of the association between the donors and recipients angiotensin-converting enzyme insertion/deletion gene polymorphism and the acute renal allograft rejection. J. Nephropathol..

[30] Bertoncello N., Moreira R.P., Arita D.Y., Aragão D.S., Watanabe I.K., Dantas P.S., Santos R., Mattar-Rosa R., Yokota R., Cunha T.S. (2015). Diabetic Nephropathy Induced by Increased Ace Gene Dosage Is Associated with High Renal Levels of Angiotensin (1-7) and Bradykinin. J. Diabetes Res..

[31] de Alcantara Santos R., Guzzoni V., Silva K.A.S., Aragão D.S., de Paula Vieira R., Bertoncello N., Schor N., Aimbire F., Casarini D.E., Cunha T.S. (2021). Resistance exercise shifts the balance of renin-angiotensin system toward ACE2/Ang 1-7 axis and reduces inflammation in the kidney of diabetic rats. Life Sci..

[32] Al-Timimi D.J., Sulieman D.M., Hussen K.R. (2014). Zinc status in type 2 diabetic patients: Relation to the progression of diabetic nephropathy. J. Clin. Diagn. Res..

[33] Nie P., Lou Y., Bai X., Zhu Y., Guo Q., Luo P., Zhang W., Li B. (2022). Influence of zinc levels and Nrf2 expression in the clinical and pathological changes in patients with diabetic nephropathy. Nutr. Diabetes.

[34] Nikoobakht M.R., Pourmand G., Allameh F., Dialameh H., Sharifi A., Hashemiaghdam A. (2014). Serum trace elements before and 3 months after renal transplantation in kidney recipients: An Iranian study. Indian J. Transplant..

[35] Muralidhara K., Kannan S., Ahamed I., Kishore K., Vincent L., Hegde N. (2021). A prospective observational study on the traditional and novel risk factors associated with post-transplant diabetes mellitus in renal transplant recipients. Biomedicine.

[36] Schüler R., Osterhoff M.A., Frahnow T., Möhlig M., Spranger J., Stefanovski D., Bergman R.N., Xu L., Seltmann A.C., Kabisch S. (2017). Dietary Fat Intake Modulates Effects of a Frequent ACE Gene Variant on Glucose Tolerance with Association to Type 2 Diabetes. Sci. Rep..

[37] ALkafaji M.J.M., Shehab A.F., Omair H.A. (2023). ACE Polymorphisms Rs (4343) and Its Association with Ace Enzyme in Some of Patients with Diabetic Nephropathy in Some Population of Salah Al-Din Governorate in Iraq. Pak. Heart J..

[38] Raza S.T., Abbas S., Siddiqi Z., Mahdi F. (2017). Association between *ACE* (rs4646994), *FABP2* (rs1799883), *MTHFR* (rs1801133), *FTO* (rs9939609) Genes Polymorphism and Type 2 Diabetes with Dyslipidemia. Int. J. Mol. Cell. Med..

[39] Zhao Y., Zhu R., Wang D., Liu X. (2019). Genetics of diabetic neuropathy: Systematic review, meta-analysis and trial sequential analysis. Ann. Clin. Transl. Neurol..

[40] Bayoumy N., El-Shabrawi M.M., Leheta O.F., Omar H.H. (2022). ACE (rs4646994) and MTHFR (rs1801133) single nucleotide polymorphisms in type 2 Diabetes Mellitus patients with dyslipidemia. Rom. J. Diabetes Nutr. Metab. Dis..

[41] Sembach F.E., Fink L.N., Johansen T., Boland B.B., Secher T., Thrane S.T., Nielsen J.C., Fosgerau K., Vrang N., Jelsing J. (2019). Impact of sex on diabetic nephropathy and the renal transcriptome in UNx db/db C57BLKS mice. Physiol. Rep..

[42] Duan J., Wang C., Liu D., Qiao Y., Pan S., Jiang D., Zhao Z., Liang L., Tian F., Yu P. (2019). Prevalence and risk factors of chronic kidney disease and diabetic kidney disease in Chinese rural residents: A cross-sectional survey. Sci. Rep..

[43] Hussain S., Jamali M.C., Habib A., Hussain M.S., Akhtar M., Najmi A.K. (2021). Diabetic kidney disease: An overview of prevalence, risk factors, and biomarkers. Clin. Epidemiol. Glob. Health.

[44] Selmi A., Aydi R., Kammoun O., Bougatef H., Bougatef A., Miled N., Alghamdi O.A., Kammoun M. (2021). Synthesis, crystal structure, molecular docking studies and biological evaluation of aryl substituted dihydroisoquinoline imines as a potent angiotensin converting enzyme inhibitor. J. Mol. Struct..

[45] Dey T.K., Chatterjee R., Mandal R.S., Roychoudhury A., Paul D., Roy S., Pateiro M., Das A.K., Lorenzo J.M., Dhar P. (2021). ACE Inhibitory Peptides from *Bellamya bengalensis* Protein Hydrolysates: In Vitro and In Silico Molecular Assessment. Processes.

[46] Januszewicz A., Januszewicz W., Szczepańska-Sadowska E., Sznajderman M. (2007). Nadciśnienie Tętnicze [Hypertension].

