# Supplementary material for: The Role of Angiotensin-Converting Enzyme (ACE) Polymorphisms in the Risk of Development and Treatment of Diabetic Nephropathy
Source: J Clin Med. 2024 Feb 8;13(4):995. doi: 10.3390/jcm13040995 (PMC10889548; doi:10.3390/jcm13040995)
Supplement: Supplementary file 1 [file jcm-13-00995-s001.zip › jcm-2831450-supplementary.pdf]

Supplementary materials

Questionnaire S1: Sample of a questionnaire conducted among people suffering from diabetic nephropathy.

PART 1. PRELIMINARY INFORMATION

Sex [W/M]	City of residence	Age [years]	Height [cm]	Weight [kg]

QUESTION	ANSWER
Do you suffer from diabetes? If so, what type?	
Do you suffer from chronic diseases other than diabetes? If so, which ones?	
Do you suffer from diabetic nephropathy?	
Have you ever been or are you undergoing dialysis?	
Have you ever received a kidney transplant?	

PART 2. LIFESTYLE

Do you play sports? How many hours per week?	
Do you smoke cigarettes? How many years? How many packs a day?	
Do you consume alcohol? In what amount? How often?	

Do you take medications/dietary supplements? Which medications or dietary supplements?

PART 3. DISEASES IN THE FAMILY

Do your parents suffer from diabetes?	
Do your siblings suffer from diabetes?	
Do your parents suffer from diabetic nephropathy?	
Do your siblings suffer from diabetic nephropathy?	

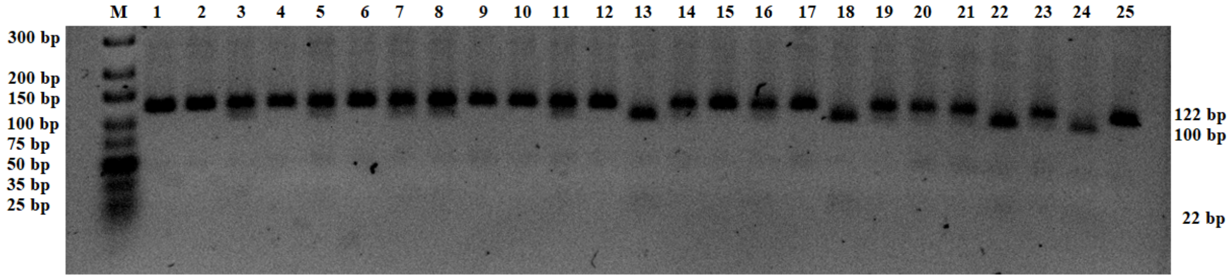

Figure S1. Example of electropherogram for rs4343 (*ACE*).
M – marker ladder; 1, 2, 4, 6, 9, 10, 12, 15, 17, 25 – G/G genotype; 3, 5, 7, 8, 11, 14, 16, 19, 20, 21, 23 – G/A genotype; 13, 18, 22, 24 – A/A genotype

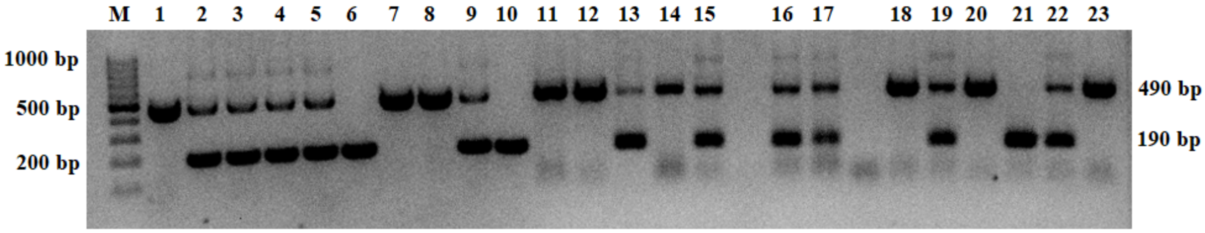

Figure S2. Example of electropherogram for rs4646994 (*ACE*).
M – marker ladder; 1, 7, 8, 11, 12, 14, 18, 20, 23 – I/I genotype; 2, 3, 4, 5, 9, 13, 15, 16, 17, 19, 22 – I/D genotype; 6, 10, 21 – D/D genotype

A.

N domain of ACE with ramipril

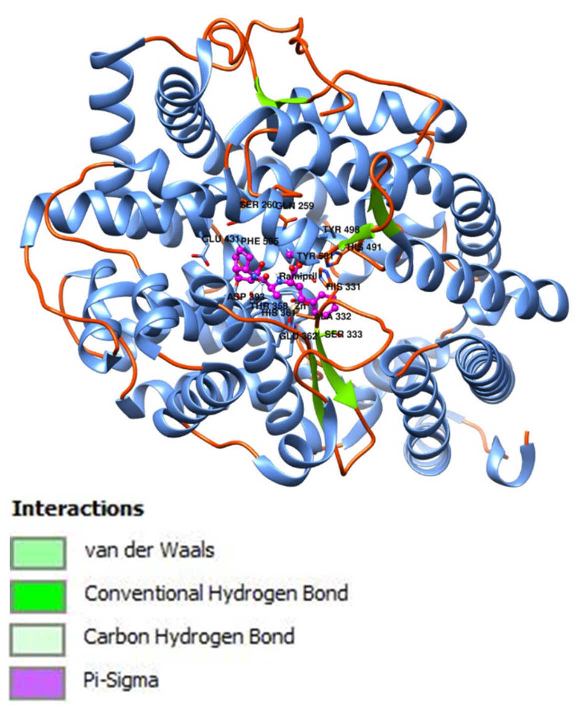

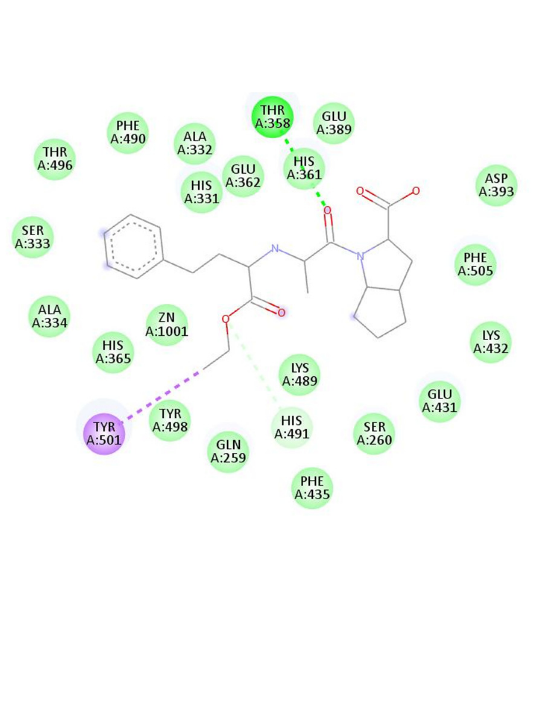

B.

C domain of ACE with ramipril

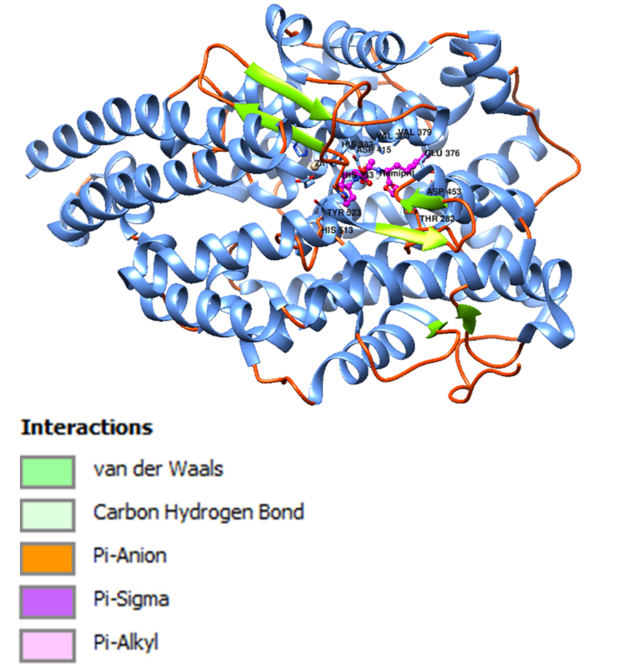

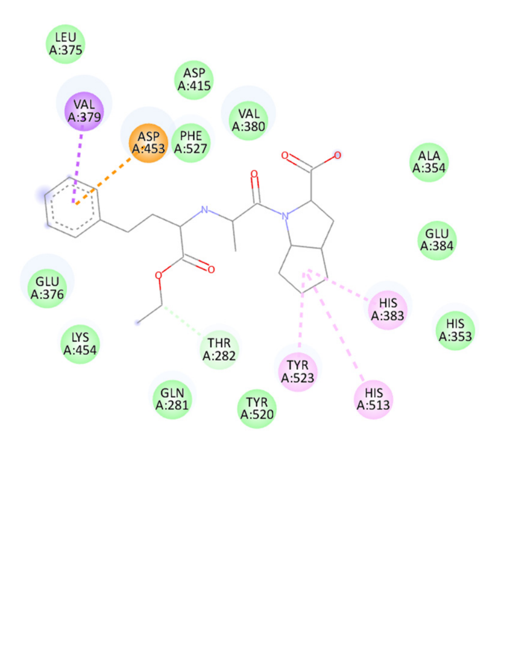

Figure S3. A, B. The interaction between N and C domains of ACE and ramipril.

A.

N domain of ACE with benazepril

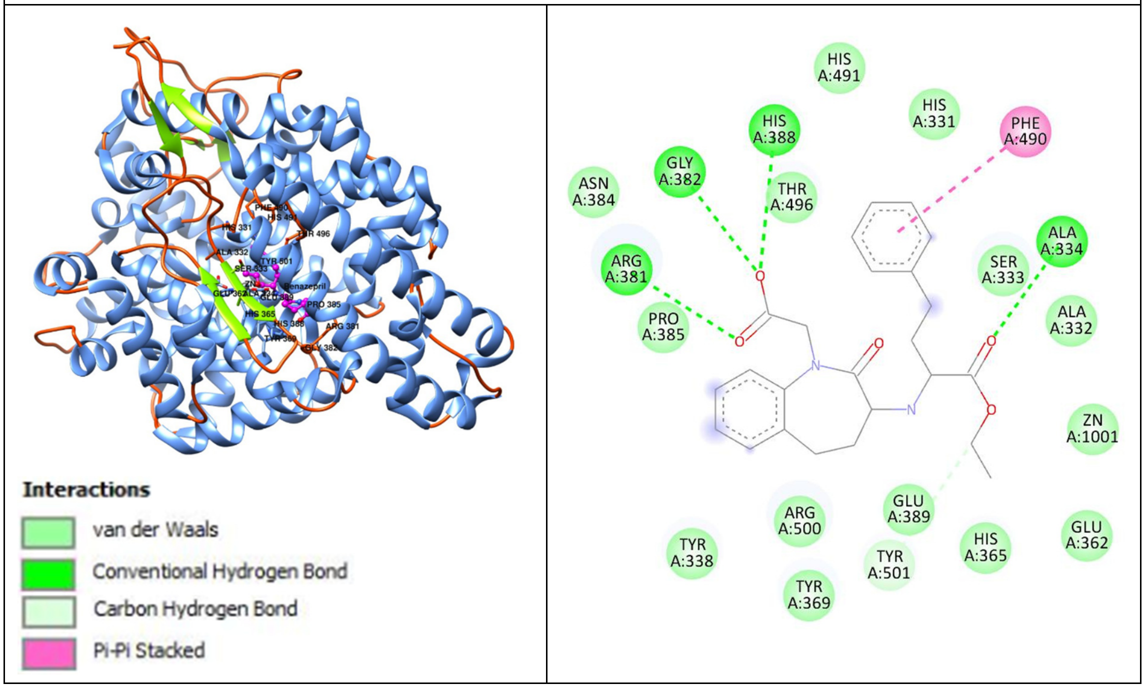

B.

C domain of ACE with benazepril

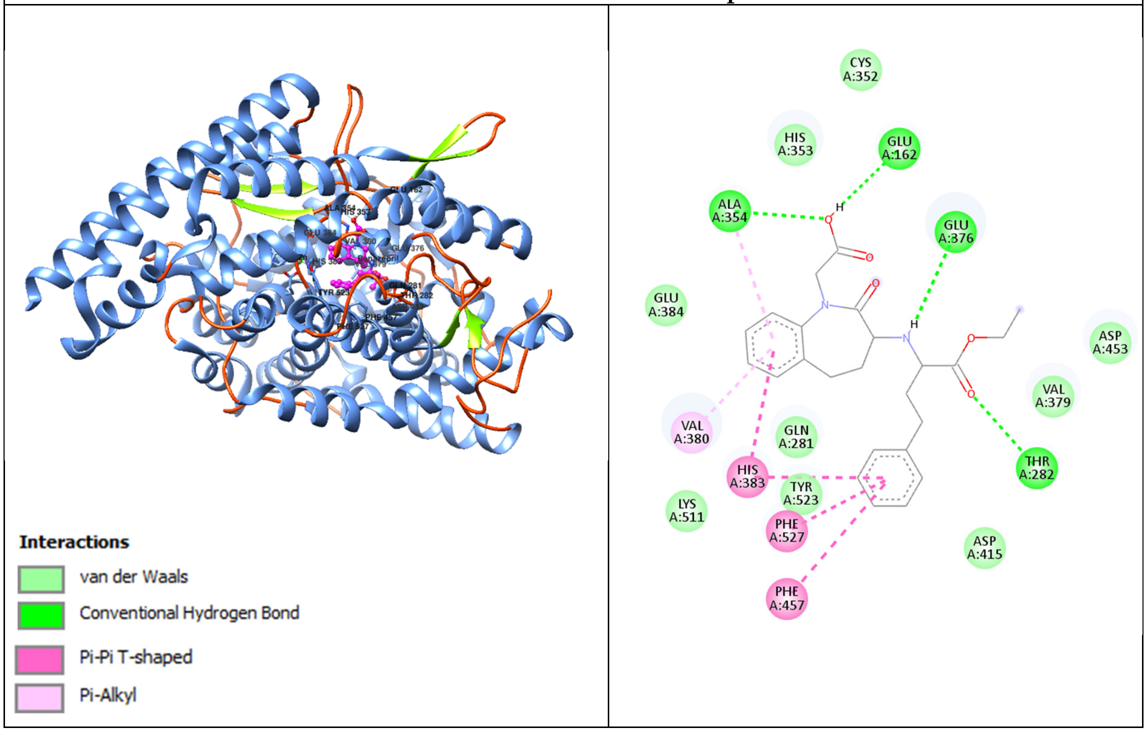

Figure S4. A, B. The interaction between N and C domains of ACE and benazepril.

A.

N domain of ACE with lisinopril

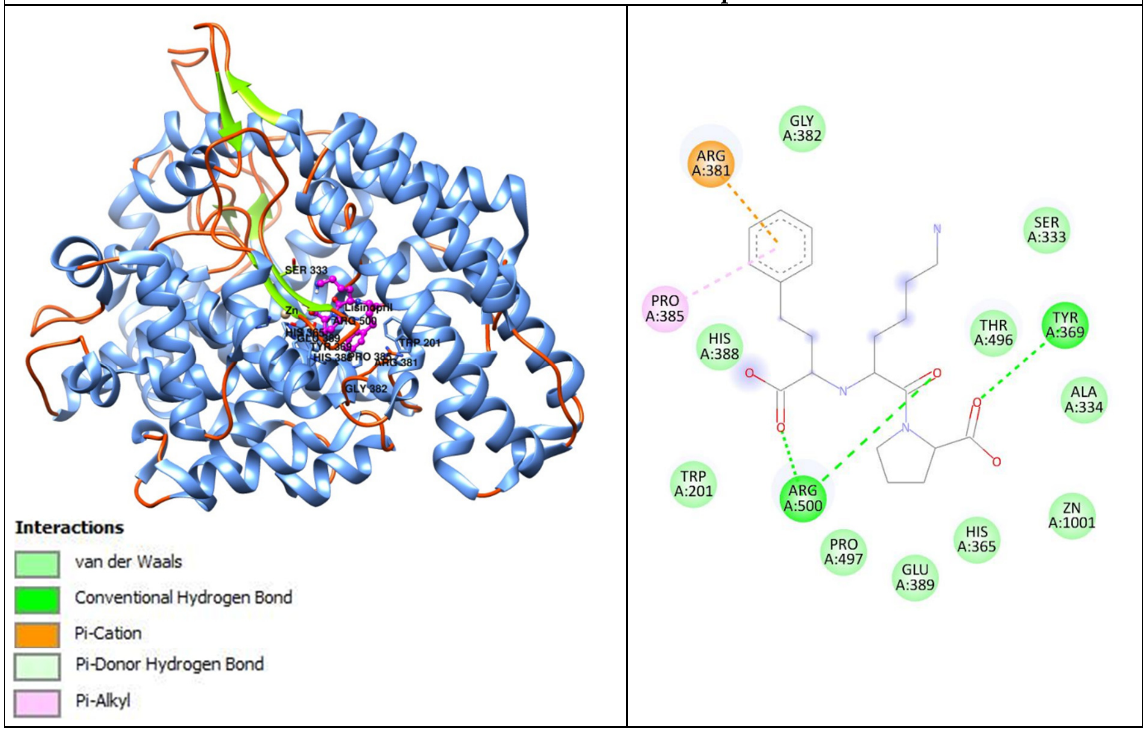

B.

C domain of ACE with lisinopril

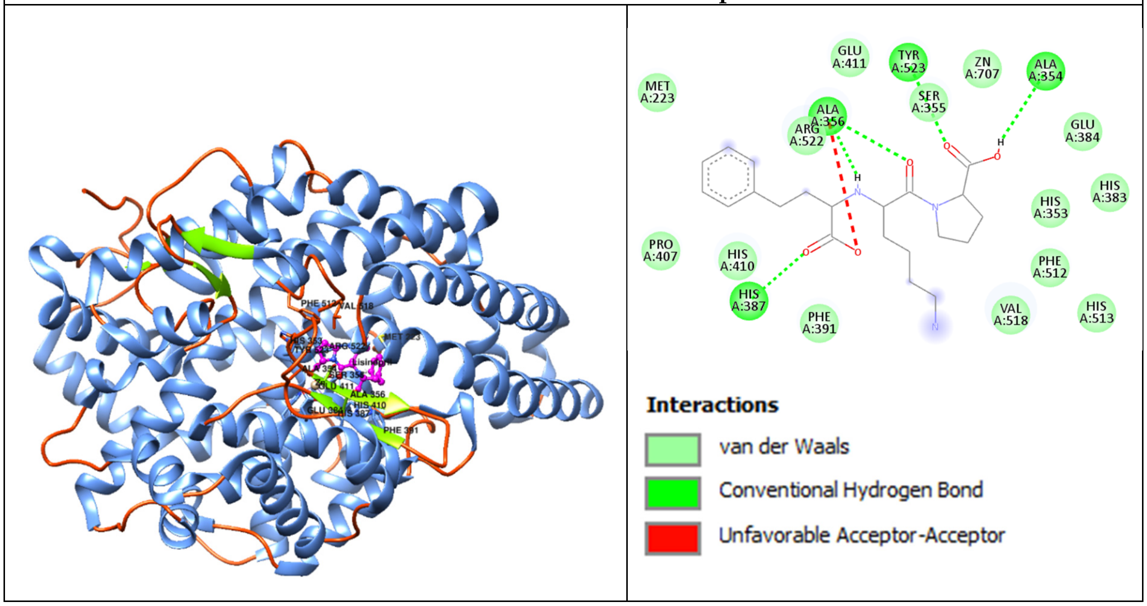

Figure S5. A, B. The interaction between N and C domains of ACE and lisinopril.
